# Conditional Poisson models: a flexible alternative to conditional logistic case cross-over analysis

**DOI:** 10.1186/1471-2288-14-122

**Published:** 2014-11-24

**Authors:** Ben G Armstrong, Antonio Gasparrini, Aurelio Tobias

**Affiliations:** Department of Social and Environmental Health Research, London School of Hygiene and Tropical Medicine (LSHTM), 15-17 Tavistock Place, London, WC1H 9SH UK; Department of Medical Statistics, London School of Hygiene and Tropical Medicine (LSHTM), Keppel Street, London, WC1E 7HT UK; Institute of Environmental Assessment and Water Research (IDAEA), Spanish Council for Scientific Research (CSIC), C/Jordi Girona 18-26, 08031 Barcelona, Spain

**Keywords:** Statistics, Conditional distributions, Poisson regression, Time series regression, Environment

## Abstract

**Background:**

The time stratified case cross-over approach is a popular alternative to conventional time series regression for analysing associations between time series of environmental exposures (air pollution, weather) and counts of health outcomes. These are almost always analyzed using conditional logistic regression on data expanded to case–control (case crossover) format, but this has some limitations. In particular adjusting for overdispersion and auto-correlation in the counts is not possible. It has been established that a Poisson model for counts with stratum indicators gives identical estimates to those from conditional logistic regression and does not have these limitations, but it is little used, probably because of the overheads in estimating many stratum parameters.

**Methods:**

The conditional Poisson model avoids estimating stratum parameters by conditioning on the total event count in each stratum, thus simplifying the computing and increasing the number of strata for which fitting is feasible compared with the standard unconditional Poisson model. Unlike the conditional logistic model, the conditional Poisson model does not require expanding the data, and can adjust for overdispersion and auto-correlation. It is available in Stata, R, and other packages.

**Results:**

By applying to some real data and using simulations, we demonstrate that conditional Poisson models were simpler to code and shorter to run than are conditional logistic analyses and can be fitted to larger data sets than possible with standard Poisson models. Allowing for overdispersion or autocorrelation was possible with the conditional Poisson model but when not required this model gave identical estimates to those from conditional logistic regression.

**Conclusions:**

Conditional Poisson regression models provide an alternative to case crossover analysis of stratified time series data with some advantages. The conditional Poisson model can also be used in other contexts in which primary control for confounding is by fine stratification.

**Electronic supplementary material:**

The online version of this article (doi:10.1186/1471-2288-14-122) contains supplementary material, which is available to authorized users.

## Background

Case crossover analysis is widely used to estimate acute associations of pollutants or other time-varying exposures with mortality or other health outcome from daily time series data from a community. Virtually all recent applications have used the more robust time stratified variant, with strata being months or day-of-week within month [1]. We focus on that variant. The strata are used to control for slow or regular (e.g. day-of-week) changes in underlying risk which might confound associations with the exposure of interest. In most applications, and those which we focus on here, pollution measurements are available only for a city or at least district, so are not unique to each individual. We call this an aggregated exposure case crossover study in contrast to an individual exposure study where exposure series are distinct for each individual. With aggregate exposures the original data are a time series of counts and environmental variables.

The standard analysis of case crossover studies is by conditional logistic regression on an expanded data set, in which for every death occurring on a day with at least one death, the day of death is entered as a “case” and other days in the same stratum as “controls” [1]. However, this is somewhat computationally intensive, and cannot allow for overdispersion or auto-correlation in the original counts, which can distort estimates. It has been established that a Poisson model for counts with stratum indicators gives identical estimates and can allow for these phenomena [2], but it is little used, probably because of the overheads in estimating many stratum parameters.

This paper describes the conditional Poisson model and demonstrates its use to simplify analysis and/or relax the assumptions of the conditional logistic regression analysis conventionally used for case cross-over studies. The aim is to give a heuristic and practical guide to the epidemiological analyst rather than a rigorous statistical exposition, for which references are given. We also compare the conditional Poisson model with the conditional logistic and also the unconditional Poisson models applied to an example dataset and some simulated data. In the discussion section we also briefly review applications of the conditional Poisson model other than for case cross-over studies.

## Methods

Our main purpose is to describe the conditional Poisson model, but before doing this we introduce the illustrative data and terminology, and briefly review the conditional logistic regression and unconditional Poisson regression formulations for case cross-over studies.

### Illustrative data

To aid understanding the models we describe their application to a study of daily air ozone pollution in relation to counts of deaths from London from 2002–6, using data previously published [3]. Primary confounder control is by stratifying time by month and day-of-week, a typical case crossover approach. A summary of the data is given in Table 1. We illustrate each method discussed using these data, which are also provided with R and Stata code reproducing the results in Additional file 1 and Additional file 2.

**Table 1:** **Description of example daily data: London 2002-2006**

Variable	Mean	Mimimum	Maximum
Date (YMD)		2002.1.1	2006.12.31
Mean temperature	11.7	-1.4	28.2
Mean ozone	34.8	18.3	119.2
Number of deaths (all cause)	149.5	99	280
Strata: year X month X day-of-week		2002.1.tues	2006.12.sun

### Notation common to all model descriptions

We suppose that data are available on counts Y_i_ of deaths (or any adverse health outcomes), a (row) vector **x**_i_ of variables of interest (here air pollution concentration) and covariates (here temperature) pertaining to each day i. The confounder control time strata (month and day-of-week) are denoted s = 1,…,S.

### Conditional logistic model for case cross-over data

Since the model formulation is standard and can be found elsewhere [2], only a summary is given here. Data are expanded to include each case and all other days in the stratum as if a matched set in a case–control study or risk set in Cox regression. Thus if there are k deaths in a stratum, the stratum data must appear k times in the expanded data set. If there are on average K deaths in a stratum, the dataset size will be multiplied by K.

With this expanded data and the notation described above, the conditional logistic model can be written
1

Where D_1,s_ is the event that the death in stratum s occurs on day i, β is a row vector of parameters, and superscript T denotes transpose.

The data duplication is reduced (say “semi-expanded”) if there are multiple deaths on the same day by multiplying the likelihood contribution from that day by the number of deaths on the case day (weighting). However, even in the semi-expanded form strata with deaths on more than one day must be repeated in the data as many times are there are days with cases, with different “case” days each time replicated.

Excerpts from the London data in the original count and semi-expanded case crossover format are presented in Tables 2 and 3. In the semi-expanded format each day is repeated four (or five) times, once as a “case” day and three (or four) times as a control day.

**Table 2:** **Excerpt from example daily data in original format**

Stratum	Date	Ozone	Temp-erature	n. of deaths
2002 1 Sun	06 jan 2002	2.4	7.1	198
2002 1 Sun	13 jan 2002	17.6	8.2	204
2002 1 Sun	20 jan 2002	49.9	8.9	167
2002 1 Sun	27 jan 2002	42.5	10.5	169
2002 1 Mon	07 jan 2002	4.1	5.2	180
. . . .

**Table 3:** **Excerpt from example data in semi-expanded format for case crossover conditional logistic analysis**

Stratum	Case-con set	Date	Ozone	Temp-erature	Case day	Weight
2002 1 Sun	2002 1 Sun 1	06 jan 2002	2.4	7.1	1	198
2002 1 Sun	2002 1 Sun 1	13 jan 2002	17.6	8.2	0	198
2002 1 Sun	2002 1 Sun 1	20 jan 2002	49.9	8.9	0	198
2002 1 Sun	2002 1 Sun 1	27 jan 2002	42.5	10.5	0	198
2002 1 Sun	2002 1 Sun 2	06 jan 2002	2.4	7.1	0	204
2002 1 Sun	2002 1 Sun 2	13 jan 2002	17.6	8.2	1	204
2002 1 Sun	2002 1 Sun 2	20 jan 2002	49.9	8.9	0	204
2002 1 Sun	2002 1 Sun 2	27 jan 2002	42.5	10.5	0	204
2002 1 Sun	2002 1 Sun 3	06 jan 2002	2.4	7.1	0	167
2002 1 Sun	2002 1 Sun 3	13 jan 2002	17.6	8.2	0	167
2002 1 Sun	2002 1 Sun 3	20 jan 2002	49.9	8.9	1	167
2002 1 Sun	2002 1 Sun 3	27 jan 2002	42.5	10.5	0	167
2002 1 Sun	2002 1 Sun 4	06 jan 2002	2.4	7.1	0	169
2002 1 Sun	2002 1 Sun 4	13 jan 2002	17.6	8.2	0	169
2002 1 Sun	2002 1 Sun 4	20 jan 2002	49.9	8.9	0	169
2002 1 Sun	2002 1 Sun 4	27 jan 2002	42.5	10.5	1	169
2002 1 Mon	2002 1 Mon 1	07 jan 2002	4.1	5.2	1	180
2002 1 Mon	2002 1 Mon 1	14 jan 2002	18.7	9.3	0	180
2002 1 Mon	2002 1 Mon 1	21 jan 2002	38.1	10.8	0	180
2002 1 Mon	2002 1 Mon 1	28 jan 2002	56.1	10.3	0	180
. . . .						

### The unconditional Poisson regression model

It has been shown that a standard (unconditional) Poisson model applied to data in the original time series format (top Table 2) with indicator variables for strata give identical estimates and inference to conditional logistic regression on expanded data – the two models are equivalent [2, 4]. The association of pollution with mortality can be thought to be inferred from the extent to which WITHIN STRATA daily death counts are explained by daily exposure concentrations. Because it provides a familiar starting point from which we can describe the conditional Poisson regression model we describe this model algebraically here.

Because control of factors changing across strata is no longer achieved by design, in addition to the regressors *x*_*i*_ we also include stratum indicator variables (a vector *z*_*i*_):
2

It helps understand the conditional variant of this model to re-write the term *α*^*T*^*z*_*i*_ as α_s_ where day i falls in stratum s (thus vector α = (α_1_,…, α_S_)) . Then the model is
3

### The conditional Poisson regression model

The conditional Poisson model is the same as model (3), except that instead of the parameters {α_s_} being estimated they are “conditioned out”, by conditioning on the sum of events  in each stratum. Technically, the conditional Poisson model is actually a multinomial model, with
4

However, describing it as a conditional Poisson model emphasizes its connections with the Poisson model and has proved convenient in formulating algorithms for packages to fit the parameters, so it is generally implemented under the conditional Poisson name. Where both can be fit, the conditional Poisson model gives identical estimates and inferences to the unconditional Poisson model and hence to the conditional logistic model (illustrated in the Results section).

The conditional Poisson model was first proposed in the econometrics literature, illustrated by a study of the dependence of annual number of patents registered by companies on their R&D expenditure [5]. It has been proposed for the self-controlled case series design in the first place for vaccine safety studies in a series of papers by Farrington and co-workers [6–8]. In this literature “exposure” typically varies between study subjects as well as over time, but a special case is where many subjects share the same exposure series, as in a typical case crossover study [9]. We are not aware of published use of the model for environmental stratified time series analyses, where the overwhelming preponderance is of conditional logistic analyses in a case crossover formulation.

The authors are familiar with implementations of the conditional Poisson model in Stata (xtpoisson with fe option) and in R (gnm with eliminate option). Examples of using these two implementations are given in Additional file 1. Strata that have no cases may be dropped, because they do not contribute to the likelihood. The EPICURE AMFIT package [10] implements the conditional Poisson model for stratified survival data under the label background stratified Poisson and this has been used quite extensively in studies of cancer effects of ionizing radiation. Richardson [11] comments that the AMFIT implementation has an unnecessary limitation in the number of strata, and proposed a method without that limitation using SAS procedure nlp or mlmixed. Xu [12] presents an approach to fit conditional Poisson models in SAS, but as this is effectively by re-formulating as a conditional logistic model we class this a conditional logistic formulation (discussed below). Many packages have programs that fit multinomial models, but these do not allow exposures *x* to vary within in each stratum *s* (e.g. pollution to vary within strata), so they cannot be used as an alternative for case crossover analyses or others that concern us here.

The conditional Poisson model, like the unconditional Poisson and conditional logistic formulations, can incorporate potentially confounding covariates not homogeneous within strata for example temperature (if air pollution is the focus). All the models can also explore modification of associations of exposure with outcomes by either such covariates or those homogeneous in strata. In the case crossover context, modifiers may be individual (e.g. age) or in multi-city studies ecological (city-level). Analyses of multi-city studies may be single-step (pooling all strata across cities) as well as the conventional multi-step (city-specific at step 1, meta-analysis at step 2). The simplicity of the conditional Poisson formulation makes the single step approach straightforward to apply (simply pool all cities into one dataset and make the strata by city as well as month and day-of-week). However, the implicit assumptions of this approach (no random or systematic between-city effects) would need investigating. A single-step analysis is particularly attractive when exposure series are available for small areas within cities.

The original event counts may have variation greater than that predicted by a Poisson distribution, so be “overdispersed” in a Poisson model. This overdispersion is not apparent in a conditional logistic analysis because in each “case–control” set in the expanded data outcomes are binary (0 or 1) for which overdispersion has no meaning. However, the assumption of independence between case–control sets in a conditional logistic model implicitly assumes no overdispersion of counts. If the binary outcomes (in the case crossover formulation) are clustered by day, then the variance of observed daily counts around the value predicted from that model will be overdispersed Poisson [2]. Where there is such overdispersion in counts a conditional logistic regression will therefore underestimate uncertainty in estimated coefficients.

Like the unconditional Poisson model with strata, the conditional model can be extended to a quasi-Poisson (overdispersed Poisson) variant, in which scale over-dispersion within strata is allowed for. In either case the over-dispersion ψ is best estimated from the Pearson chi-squared statistic, though neither this nor other estimates are consistent when data are sparse (few events per stratum) [13]. Quasi-Poisson is an option in the R implementation, and can be implemented in Stata with some post-processing (see Additional file 1).

Similarly, the methods discussed by Brumback [14] for allowing for autocorrelation for count time series in general can be applied to the conditional as well an unconditional Poisson models. We are not aware of any off the self-software implementation but ad hoc implementations in Stata and R are described in additional file 1. As with overdispersion, it is sometimes thought that a case crossover analysis, especially if stratified by day of week, is not affected by autocorrelation. However, the case crossover formulation assumes that observations (in the expanded data format) are independent both within and across strata – an assumption that is violated if there is residual autocorrelation in counts.

The Poisson models can also accommodate studies where rate denominators (durations of time intervals or numbers of subjects at risk) vary between study units (“days”) by using an appropriate offset. Residual and influence analysis is also possible with the Poisson models.

The conditional logistic formulation does not easily allow any of these extensions apart from the incorporation of covariates.

### Comparing processor time taken in fitting each model

To compare processor time taken to fit each of the three models described above we simulated datasets with a range of sizes, corresponding to possible scenarios. For each scenario we simulated ten years of daily data. Baseline mortality rates of 1,10, and 100 deaths/day represented small, medium, and large cities. Three more data-sets included multiples of this baseline number of days to illustrate multi-city or multi-area studies analysed in one stage. Outcome counts were generated to follow a Poisson distribution with mean given by the exponent of a linear sum of seven covariates (exposures and confounders). The covariates were distributed as multivariate normal, mutually correlated at r = 0.25, and scaled so that one standard deviation of each covariate was associated with a rate ratio of 1.05. Two types of case cross-over stratification were considered: by month and day-of-week, as described above, and by month only.

## Results

Using our illustrative data set, we estimated the coefficient for ozone (per 10 ug/m3) using each of the three models described above (conditional logistic, unconditional Poisson and conditional Poisson), controlling for temperature, rather crudely to simplify the illustration, as a linear term at lag 0. The estimates were, as expected, identical whether analysed using standard conditional logistic, unconditional Poisson or conditional Poisson models (Table 4). Programming was simpler for the Poisson models than for the conditional logistic formulation because no data expansion was necessary (Additional file 1). The unconditional Poisson model fitted coefficients for the 420-1 = 419 extra coefficients for the stratum indicator variables, giving somewhat cumbersome output but in this data set not a serious increase in computation time.

**Table 4:** **Fitting the models to the London 2002–6 data**

Model	Coefficient (9%% estimate)	Overdispersion	N. of coefficients
Conditional logistic	0.34% (0.03,0.65)	1	2
Unconditional poisson	0.34% (0.03,0.65)	1	421
Conditional poisson	0.34% (0.03,0.65)	1	2
+ overdispersion	0.34% (-0.03,0.70)	1.37	2
+ auto-correlation	0.27% (-0.05,0.58)	1	3

Scale overdispersion, estimable using the quasi-Poisson models, was ψ = 1.37 (probably large due to failure to control well for temperature), and the CI for the coefficient estimated by quasi-Poisson consequently wider than that estimated by Poisson or conditional logistic model (-0.03,0.70 compared to 0.03,0.65). Allowing for first order autocorrelation (using the method of Brumback) changed the estimated ozone coefficient from 0.34% (0.03,0.65) to 0.27% (-0.05,0.58).

The table in Additional file 3 summarises the practicalities of using these three types of model and computer time for a range of hypothetical data sets obtained by simulation. Where all models can be fit they gave identical estimated coefficients and standard errors, as we saw in the example data and anticipate from theory [2, 15]. With large number S of strata (500–1000 depending on hardware and software) fitting the unconditional Poisson model becomes impossible because it depends on inverting a matrix somewhat larger than S squared. The conditional Poisson model was faster than the unconditional Poisson or conditional logistic formulation, though times for the latter were not prohibitively long unless the numbers of strata were very large indeed, or fitting the model is embedded in an iterative algorithm, for example in a Bayesian model fit by MCMC [16, 17].

## Discussion

The conditional Poisson model is a little known alternative to the conventional conditional logistic model option for analysis of time stratified counts in a case crossover formulation, with some attractive features. In particular the conditional Poisson model can allow for overdispersion, autocorrelation and varying rate denominators, which are not options for conditional logistic regression. It also simplifies coding and reduces processor time.

We have considered aggregated exposure time series data, which are the most common application of case crossover analyses. Where exposure series are individual or close to it and outcomes occur only once in an individual conditional Poisson offers little advantage, leaving the conditional logistic case crossover formulation the natural choice.

The description of the conditional Poisson model as a “fixed effect” model suggests the possibility of fitting a random stratum effect (mixed model) rather than fixed effect. If the variable of interest (say “exposure”) varies over strata then the coefficient of exposure in the random effects model will have greater precision that that from the fixed effect (i.e. conditional Poisson) model. However this is bought at the expense of the assumption on distributional form (typically Gaussian) for the random effect, and some degree of vulnerability to confounding by between- as well as within- stratum variation in risk factors (for example seasonal). We urge caution in assuming random stratum effects for this reason.

### Application of the conditional Poisson model outside of case cross-over studies

There are several applications of conditional Poisson models other than as an alternative to conventional conditional logistic case crossover analysis, which we mention briefly in this and the next three paragraphs.

One use is in injury research for matched cohort studies [18]. One such application which comes close to the stratified time series context sought to identify whether the imposition of 20 mph speed restrictions on London residential streets reduced injuries [19]. The data comprised dated injury records referenced by street segment and dates at which speed restrictions were imposed. This could be thought of as a multiple interrupted time series study, with each street segment (of approximately 300,000) providing multiple time series of about 900,000 injuries in total. The pre- and post-intervention periods contributed the exposed and unexposed days. Other factors changing over time assumed to have the same affect across London were controlled by covariate terms in the conditional Poisson model, while conditioning on road segment.

Another potential area of application is where the aim is to identify if adverse event counts vary over time in concert with (numerical) exposure in multiple short series in small areas. For example Tonne [20] considered the association of changes over four years in exposure to air pollutants with changes in hospitalization counts (about 400,000 admissions) in 638 small areas (electoral wards) in London. In the original analysis the time interval was aggregated to just two sub-periods, so as to allow a binomial regression, which is a special case of multinomial and thus as noted above is equivalent to conditional Poisson regression. Conditional Poisson analysis could have provided more power by obviating the need to aggregate into two periods and retaining the original four years as separate study units.

The last range of applications we will mention is to panel studies of count outcomes. Much of the econometric literature on conditional Poisson models (and the Stata documentation) is framed in this context, in which “panels” of repeated observations of counts comprise the strata. Many of the designs we have described above can be formulated in terms of panels, but more directly we might envisage explicit epidemiological panel studies using conditional Poisson regression. These generally comprise panels of patients with exposures and outcomes repeated over several time periods, making the context similar to that considered in Farrington’s papers under the self controlled case series label. If the outcomes are counts (e.g. numbers of inhaler uses in a day in asthmatics), a conditional Poisson model seems natural, and is indeed proposed by Farrington and colleagues [21].

## Conclusions

The conditional Poisson model offers an alternative to the conditional logistic model with expanded data for time stratified case crossover and related analysis, offering extra flexibility by allowing for overdispersion, autocorrelation, and varying rate denominators.

## Electronic supplementary material

Additional file 1: **R and Stata code for conditional Poission analysis.** This is a pdf document. (DOCX 27 KB)

Additional file 2: **London 2002–6 daily mortality data set for use in illustrative analyses.** This is a Stata dataset (.dta). (ZIP 23 KB)

Additional file 3: **Computational Issues in Conditional Poisson and Related Models.** This large format table is a pdf document. (DOCX 23 KB)
