# Supplementary material for: Conditional Poisson models: a flexible alternative to conditional logistic case cross-over analysis
Source: BMC Med Res Methodol. 2014 Nov 24;14:122. doi: 10.1186/1471-2288-14-122 (PMC4280686; doi:10.1186/1471-2288-14-122)
Supplement: Supplementary file 1 — Additional file 1: R and Stata code for conditional Poission analysis. This is a pdf document. (DOCX 27 KB) [file 12874_2014_1140_MOESM1_ESM.docx]

## Conditional Poisson models: a flexible alternative to conditional logisitic case cross-over analysis

## Additional file 1. R and Stata code for conditional Poission analysis

Variable name notation:

t: time sequence

x: exposure (eg pollution)

v1-v6: covariates (eg temperature spline)

y: outcome count

stratum: stratum indicator (eg for yearXmonthXday-of-week)

dur duration of time interval (if not equal)

### Stata core code:

xtset stratum

xtpoisson y v1 v2 v3 v4 v5 v6, fe

with overdispersion

xtpoisson y v1 v2 v3 v4 v5 v6, fe

xtpoisson_addOD // (defined below)

with unequal time intervals or other rate denominator :

xtpoisson y v1 v2 v3 v4 v5 v6, fe e(dur)

with Brumback autocorrelation adjustment (after code above for overdipersion)

gen devreslag1=_xtp_devianceres[_n-1]

xtset stratum

xtpoisson y x1-x6 devreslag1 , fe

Stata progam to correct estimates for overdispersion

******************************************************************************

*** PROGRAM TO CORRECT ESTIMATES FOR OVERDISPERSION AFTER USING XTPOISSON, FE

capture program drop xtpoisson_addOD

program def xtpoisson_addOD, eclass

dis _n(1) "Estimate and standard errors corrected for over-dipersion"

tempvar ppred nonmissxY stratumsumY stratumsumpred pred x2

qui predict `ppred', nu0 // GIVES PRED COUNT WITHOUT STRATUM EFFECT

local Y `e(depvar)'

local i `e(ivar)' // STRATUM INDEX VARIABLE

local dfres=e(N)-e(df_m)-e(N_g) // DF OF THE RESIDUALS

qui gen `nonmissxY'=`Y'*(`ppred'!=.)

qui egen `stratumsumY'=sum(`nonmissxY'), by(`i')

qui egen `stratumsumpred'=sum(`ppred'), by(`i')

qui gen `pred'=`ppred'*`stratumsumY'/`stratumsumpred' // RESCALES PRED COUNTS TO MATCH STRATUM SUMS

qui gen `x2'=(`Y'-`pred')^2/(`pred')

qui summ `x2'

local dispers=r(sum)/`dfres'

dis "df: `dfres' ; pearson x2:" %8.1f r(sum) " ; dispersion: " %8.2f `dispers'

matrix B=get(_b)

matrix V=get(VCE)

matrix corrV=V*`dispers'

ereturn scalar dispers=`dispers'

ereturn post B corrV

ereturn display

*STORE PREDICTED COUNTS AND PEARSON RESIDUALS

capture drop _xtp_pred_count

qui gen _xtp_pred_count = `pred'

capture drop _xtp_pearsonres

qui gen _xtp_pearsonres= (`x2'^.5)*sign(`Y'-`pred')

capture drop _xtp_devianceres

qui gen _xtp_devianceres= sqrt( 2*(`Y'*log(`Y'/`pred')-( `Y'-`pred') )) *sign(`Y'-`pred')

end

******************************************************************************

Full worked example in stata

*******************************************************************************

* R code to ilustrate conditional Poisson regression analysis as described in

* Armstrong et al, "Conditional Poisson models ..." [BMC Med Res Methods] 2014

* Uses data from: "Time series regression studies in environmental epidemiology"

* Bhaskaran et al International Journal of Epidemiology - 2013

* THE ANALYSIS IS AN EXERCISE ONLY. IN PARTICULAR THERE IS POOR CONTROL FOR TEMPERATURE

*

* 09 05 2014

* For any problem with this code, please contact ben.armstrong@lshtm.ac.uk

*******************************************************************************

use londondataset2002_2006, clear

* DIVIDE THE OZONE VARIABLE BY 10 SO THAT MODEL ESTIMATES REFER TO A

* "PER 10ug/m3 INCREASE" (AS PER CONVENTION)

replace ozone = ozone/10

rename ozone ozone10

label var ozone10 "Ozone level in ug/m3 divided by 10"

* CREATE YEAR X MONTH X DOW STRATUM VARIABLE

gen month=month(date)

gen year=year(date)

gen dow=dow(date)

egen stratum_YMD=group(year month dow)

*** FIT CONDITIONAL POISSON MODEL

xtset stratum_YMD

xtpoisson numdeaths ozone10 temperature, fe

*** NOW ALLOW FOR OVERDISPERSION (function xtpoisson_addOD below, used after xtpoisson)

xtpoisson_addOD

*** ADD BRUMBACK AUTOCORRELATION ADJUSTMENT (NEED TO HAVE USED xtpoisson_addOD BEFORE)

gen devreslag1=_xtp_devianceres[_n-1]

xtpoisson numdeaths devreslag1 ozone10 temperature , fe

* FINALLY ADD ALLOWANCE FOR OVERDISPERSION TO THAT FOR AUTOCORRELATION

xtpoisson_addOD

*** ILLUSTRATION OF ALLOWING FOR VARYING RATE DENOMINATORS

** FOR THIS WE HAVE IMAGINED AVAILABILITY OF A RELEVANT POPULATION MEASURE CHANGING

** AT SHORT TIME SCALES (THOUGH ARFICIALLY SPECIFIED HERE AS A CONSTANT, TO DEMONSTRATE CODE)

gen population = 3000000

xtpoisson numdeaths ozone10 temperature, exp(population) fe

** FURTHER CODE FOR UNCONDITIONAL POISSON AND CONDITIONAL LOGISTIC (CASE CROSSOVER)

** ANALYSES REPORTED IN THE TEXT

*** FIT UNCONDITIONAL POISSON MODEL

set matsize 500

glm numdeaths i.stratum_YMD ozone10 temperature , f(poisson)

*** FIT CONDITIONAL LOGISTIC MODEL

* FIRST EXPAND DATA

save temp, replace

use temp, clear

sort stratum date

gen one=1 // convenience variable

by stratum: gen origdos=sum(one) // numbers days in strata 1-4 or 1-5

by stratum: egen n_in_stratum = max(origdos)

expand n_in_stratum

sort stratum origdos

by stratum origdos: gen dos=sum(one) // distribute duplicated days across case-ref sets

gen caseday=(dos==origdos) // set indicator for case day

egen ccset=group(year month dow dos) , label

* WEIGHT OBSERVATIONS BY N OF DEATHS ON INDEX DAY

gen tempweight=numdeaths*caseday

egen weight=max(tempweight), by(ccset)

drop if weight==0

* CLOGIT ANALYSIS

clogit caseday ozone10 temperature [fweight=weight], group(ccset)

### R core code:

gnm(y ~ x + v1 + v2 + v3 + v4 + v5 + v6,

family=poisson(), data=usedata, eliminate=stratum)

with overdispersion:

gnm(y ~ x + v1 + v2 + v3 + v4 + v5 + v6,

family=quasipoisson(), data=usedata, eliminate=stratum)

with unequal time intervals or other rate denominator :

gen logdur=log(dur)

gnm(y ~ x + v1 + v2 + v3 + v4 + v5 + v6,

family=quasipoisson(), data=usedata, eliminate=stratum,

offset=logdur)

with Brumback autocorrelation adjustment

model1 <- gnm(y ~ x + v1 + v2 + v3 + v4 + v5 + v6,

family=quasipoisson(), data=usedata, eliminate=stratum)

reslag1 <- Lag(residuals(model1,type="deviance"),1)

gnm(y ~ x + v1 + v2 + v3 + v4 + v5 + v6 + reslag1,

family=quasipoisson(), data=usedata, eliminate=stratum)

Full worked example in R

###############################################################################

# R code to illustrate conditional Poisson regression analysis as described in:

# Armstrong et al, "Conditional Poisson models ...",

# [BMC Med Res Methods] 2014

#

# Uses data from:

# "Time series regression studies in environmental epidemiology", Bhaskaran et

# al International Journal of Epidemiology - 2013

#

# THE ANALYSIS IS AN EXERCISE ONLY.

# IN PARTICULAR THERE IS POOR CONTROL FOR TEMPERATURE

#

# 10 Dec 2013

# users will need to set the working directory and install the packages gnm and tsModel

# For any problem with this code, please contact ben.armstrong@lshtm.ac.uk ###############################################################################

library(foreign) # ENABLES READING THE DATA FILE, WHICH IS A STATA FORMAT

data <- read.dta("londondataset2002_2006.dta")

summary(data)

# SET THE DEFAULT ACTION FOR MISSING DATA TO na.exclude

# (MISSING EXCLUDED IN ESTIMATION BUT RE-INSERTED IN PREDICTION/RESIDUALS)

options(na.action="na.exclude")

# SCALE EXPOSURE

data$ozone10 <- data$ozone/10

# GENERATE MONTH AND YEAR

data$month <- as.factor(months(data$date))

data$year <- as.factor(format(data$date, format="%Y") )

data$dow <- as.factor(weekdays(data$date))

data$stratum <- as.factor(data$year:data$month:data$dow)

data <- data[order(data$date),]

# FIT A CONDITIONAL POISSON MODEL WITH A YEAR X MONTH X DOW STRATA

library(gnm)

modelcpr1 <- gnm(numdeaths ~ ozone10 + temperature, data=data, family=poisson,

eliminate=factor(stratum))

summary(modelcpr1)

# ALLOW FOR OVERDISPERSION

modelcpr2 <- gnm(numdeaths ~ ozone10 + temperature , data=data, family=quasipoisson,

eliminate=factor(stratum) )

summary(modelcpr2) # ANTONIO - summary NOT WORKING ON MY PC

# ADD BRUMBACK AUTOCORRELATION ADJUSTMENT

library(tsModel) # FACILITATES GETTING LAGGED VALUES'

reslag1 <- Lag(residuals(modelcpr1,type="deviance"),1)

modelcpr3 <- gnm(numdeaths ~ ozone10 + temperature + reslag1, data=data,

family=quasipoisson, eliminate=factor(stratum))

summary(modelcpr3)

# ALLOW FOR AUTOCORRELATION AND OVERDISPERSION

library(tsModel) # FACILITATES GETTING LAGGED VALUES'

reslag1 <- Lag(residuals(modelcpr1,type="deviance"),1)

modelcpr4 <- gnm(numdeaths ~ ozone10 + temperature + reslag1, data=data,

family=quasipoisson, eliminate=factor(stratum))

summary(modelcpr4)

# ILLUSTRATION OF ALLOWING FOR VARYING RATE DENOMINATORS

# FOR THIS WE HAVE IMAGINED AVAILABILITY OF A RELEVANT POPULATION MEASURE CHANGING

# AT SHORT TIME SCALES

data$population <- 3000000

logpop <- log(data$population)

modelcpr5 <- gnm(numdeaths ~ ozone10 + temperature, data=data, family=poisson , offset=logpop,

eliminate=factor(stratum))

summary(modelcpr5)

# FURTHER CODE FOR THE UNCONDITIONAL POISSON AND CONDITIONAL LOGISTIC (CASE CROSSOVER)

# ANALYSES REPORTED IN THE TEXT

# FIT UNCONDITIONAL POISSON MODEL

model_upr <- glm(numdeaths ~ ozone10 + temperature + factor(stratum),data=data,family=poisson)

summary(model_upr)

# FIT CONDITIONAL LOGISTIC MODEL

# EXPAND THE DATA IN A CASE-CROSSOVER FORMAT (AND EXCLUDE STRATA WITH 0)

# REQUIRED FUNCTION

funccmake <- function(date,cases,vars=NULL,dow) {

#

# DERIVE STRATUM VARIABLES

if(missing(dow)) dow <- ifelse(class(date)=="Date",TRUE,FALSE)

if(class(date)=="Date") {

day <- if(dow) weekdays(date) else rep(1,length(date))

month <- months(date)

year <- format(date, format="%Y")

} else {

day <- rep(1,length(date))

month <- date

year <- rep(1,length(date))

if(dow) stop("'dow' only available when 'date' is a date")

}

#

# DERIVE INDEXING VARIABLES

gfactor <- factor(day):factor(month):factor(year)

gnumber <- match(gfactor,unique(gfactor))

gindex <- lapply(1:length(date),

function(x) (1:length(date))[gnumber%in%gnumber[x]])

gstatus <- lapply(1:length(date), function(x) gindex[[x]]==x)

#

# EXPAND PREDICTORS

if(!is.null(vars)) {

varnames <- if(is.vector(vars)) deparse(substitute(vars)) else names(vars)

vars <- as.matrix(vars)

dimnames(vars) <- list(NULL,varnames)

}

#

# RESULTS

res <- data.frame(

index=unlist(gindex),

status=unlist(gstatus)+0,

stratum=rep(1:length(date),sapply(gindex,length)),

weights=rep(cases,sapply(gindex,length))

)

if(!is.null(vars)) res <- cbind(res,vars[res$index,])

#

return(res)

}

dataexp <- funccmake(data$stratum,data$numdeaths,vars=cbind(data$ozone10,data$temperature ))

dataexp <- dataexp[dataexp$weights>0,]

Xexp <- as.matrix(dataexp)[,-seq(4)]

# RUN CLR

library(survival)

timeout <- as.numeric(factor(dataexp$stratum))

timein <- timeout-0.1

model_clr <- coxph(Surv(timein,timeout,status) ~ Xexp, weights=weights, dataexp)

summary(model_clr)
