# Supplementary material for: Conditional Poisson models: a flexible alternative to conditional logistic case cross-over analysis
Source: BMC Med Res Methodol. 2014 Nov 24;14:122. doi: 10.1186/1471-2288-14-122 (PMC4280686; doi:10.1186/1471-2288-14-122)
Supplement: Supplementary file 3 — Additional file 3: Computational Issues in Conditional Poisson and Related Models. This large format table is a pdf document. (DOCX 23 KB) [file 12874_2014_1140_MOESM3_ESM.docx]

## Conditional Poisson models: a flexible alternative to conditional logisitic case cross-over analysis

**Additional file 3. Large format table: Computational Issues in Conditional Poisson and Related Models.**

|  |  |  | Model | | | | | |
| --- | --- | --- | --- | --- | --- | --- | --- | --- |
|  |  |  | Conditional Poisson Regression | | Unconditional Poisson Regression | | Conditional Logistic Regression | |
|  |  |  |  | |  | |  | |
| Extensions | overdispersion |  | Y^1^ | | Y | | N | |
|  | autocorrelation |  | Y^2^ | | Y^2^ | | N | |
|  | rate denominators |  | Y | | Y | | N | |
|  |  |  |  | |  | |  | |
| Computing time (seconds) examples (10y data) | | N of strata | Stata | R | Stata | R | Stata | R |
| Month strata | small city (1 event/day) | 120 | 0.2 | <0.1 | 0.5 | 0.5 | 3.0 | 1.6 |
|  | medium city (10 e/day) | 120 | 0.2 | <0.1 | 0.4 | 0.4 | 3.0 | 2.2 |
|  | large city (100 e/day) | 120 | 0.2 | <0.1 | 0.5 | 0.3 | 3.2 | 1.5 |
|  | 10 medium cities 1-stage analysis | 1200 | 1.0 | 0.5 | NF | 292 | 26.7 | 2605.0 |
|  | 10 small areas (0.1 e/day) 1-stage a. | 1200 | 1.0 | 0.5 | NF | 1180 | 6.6 | 39.0 |
|  | 100 v. small areas (0.01 e/day) 1-stage a. | 12000 | 5.9 | 7.1 | NF | NF | 57.3 | >3000 |
|  |  |  |  |  |  |  |  |  |
| Month X day  of week strata | small city | 480 | 0.3 | <0.1 | NF | 16.6 | 0.5 | 2.7 |
|  | medium city | 480 | 0.3 | <0.1 | NF | 13.9 | 0.5 | 0.7 |
|  | large city | 480 | 0.2 | <0.1 | NF | 10.8 | 0.5 | 0.5 |
|  | 10 medium cities 1-stage analysis | 4800 | 2.3 | 0.4 | NF | NF | 5.0 | 452.0 |
|  | 10 small areas (0.1 e/day) 1-stage a. | 4800 | 1.8 | 0.6 | NF | NF | 1.4 | 44.0 |
|  | 100 v. small areas (0.01 e/day) 1-stage a. | 48000 | 12.6 | 8.9 | NF | NF | 8.3 | >3000 |

NF = not feasible due to memory limitations

^1^ Stata requires ad hoc added code – see additional file 2

^2^  Stata and R require ad hoc added code – see additional file 2

All analyses undertaken on a Samsung Notebook NPU250U2B with Intel Core i3-2330M CPU @ 2.2 GHz with 4 Gb Ram and 64-bit OS running Windows 7 Home Premium
